# Motion artifact cancellation in NIR spectroscopy using discrete Kalman filtering

**DOI:** 10.1186/1475-925X-9-16

**Published:** 2010-03-09

**Authors:** Meltem Izzetoglu, Prabhakar Chitrapu, Scott Bunce, Banu Onaral

**Affiliations:** 1 School of Biomedical Eng, Science and Health Sys, Drexel University, Philadelphia, PA 19104, USA; 2 InterDigital Communications Corp King of Prussia, PA 19406, USA; 3 Hershey Medical Center, Penn State University, Hershey, PA 17033, USA

## Abstract

**Background:**

As a continuation of our earlier work, we present in this study a Kalman filtering based algorithm for the elimination of motion artifacts present in Near Infrared spectroscopy (NIR) measurements. Functional NIR measurements suffer from head motion especially in real world applications where movement cannot be restricted such as studies involving pilots, children, etc. Since head movement can cause fluctuations unrelated to metabolic changes in the blood due to the cognitive activity, removal of these artifacts from NIR signal is necessary for reliable assessment of cognitive activity in the brain for real life applications.

**Methods:**

Previously, we had worked on adaptive and Wiener filtering for the cancellation of motion artifacts in NIR studies. Using the same NIR data set we have collected in our previous work where different speed motion artifacts were induced on the NIR measurements we compared the results of the newly proposed Kalman filtering approach with the results of previously studied adaptive and Wiener filtering methods in terms of gains in signal to noise ratio. Here, comparisons are based on paired t-tests where data from eleven subjects are used.

**Results:**

The preliminary results in this current study revealed that the proposed Kalman filtering method provides better estimates in terms of the gain in signal to noise ratio than the classical adaptive filtering approach without the need for additional sensor measurements and results comparable to Wiener filtering but better suitable for real-time applications.

**Conclusions:**

This paper presented a novel approach based on Kalman filtering for motion artifact removal in NIR recordings. The proposed approach provides a suitable solution to the motion artifact removal problem in NIR studies by combining the advantages of the existing adaptive and Wiener filtering methods in one algorithm which allows efficient real time application with no requirement on additional sensor measurements.

## Background

Near infrared spectroscopy is an emerging technology which enables the measurement of changes in the concentration of deoxygenated hemoglobin (deoxy-Hb) and oxygenated hemoglobin (oxy-Hb) noninvasively during functional brain activation in humans [1]. The technology allows the design of portable, safe, affordable, non-invasive and negligibly intrusive monitoring systems which makes it suitable for many operations, including the monitoring of ongoing cognitive activity under routine working conditions and in the field [1-3].

Typically, an optical apparatus consists of a light source by which the tissue is irradiated and a light detector that receives the light after it has interacted with the tissue. In NIR spectroscopy, the range of light used is between 700 to 900 nm since biological tissues are relatively transparent to light in this range [1]. This is mainly due to the fact that within this so called "optical window", the absorbance of the main constituents in the human tissue such as water, oxy- and deoxy-hemoglobin is small allowing the light to penetrate the tissue. Among the main absorbers (chromophores) in the tissue, oxy- and deoxy-Hb are strongly linked to tissue oxygenation and metabolism. Fortunately, in the optical window, the absorption spectra of oxy- and deoxy-Hb remain significantly different allowing spectroscopic separation of these compounds using only a few sample wavelengths. In functional brain imaging studies, since the demand and the consumption of these main absorbers in the brain change during cognitive activity, monitoring the change in their concentrations using NIR spectroscopy provides information about brain function [1-3]. In functional NIR applications, two other variables, namely oxygen index and blood volume, are commonly used to extract information about the cognitive activities performed. They are derived from the change in the concentrations of oxy-Hb and deoxy-Hb extracted from NIR measurements using Beer-Lambert Law. (Detailed information on the calculation of oxygen index and blood volume can be found in [1,3]).

Due to many attractive atributes, NIR is an ideal candidate for monitoring cortical function in the brain while subjects are engaged in various real life or experimental tasks. However, functional NIR measurements suffer from head motion [4], especially in real world applications where movement cannot be restricted such as in studies involving pilots, children, etc. Head movement can cause fluctuations unrelated to metabolic changes in the blood due to the cognitive activity. These artifacts are often due to the loss of contact of NIR detectors with skin resulting in measurements of either the ambient light or the light emitted directly from the NIR sources. Furthermore, head movement can cause the blood to move towards or away from the measurement area causing the amount of oxygen to increase or decrease in the region of interest. Therefore, removal of motion artifacts from NIR signal is necessary for reliable assessment of cognitive activity in the brain, hence critical to its deployment as a brain monitoring technology suitable for real life applications.

In this article, we propose a new solution for the motion artifact removal from the NIR signal based on Kalman filtering. To our knowledge, adaptive filtering and Wiener filtering are the only techniques used to solve this problem [4]. Both techniques have been widely used for noise reduction in many biomedical, communication, speech processing applications [4-8]. An adaptive filter is usually a finite impulse response (FIR) filter which has an adaptation algorithm that monitors the environment with additional sensors and hardware and varies the filter transfer function according to the changing input signal's characteristics [5-8]. Like adaptive filtering, Wiener filter is an optimal filtering method in the mean square sense, however it uses the statistics of the signals involved to estimate the filter coefficients without the need for additional sensor information [5-8]. Wiener filtering in general demands stationary data and may not be applied in real time efficiently.

In our application, Kalman filtering approach overcomes the problem of using additional sensors and extra wiring requirement of the adaptive filtering. Due to its recursive nature it further allows efficient real time implementation even without stationarity requirements on the data. Results obtained by Kalman filtering achieve better signal to noise ratios (SNR) than the adaptive filtering and are comparable in SNR to Wiener filtering. The performance of the Kalman filter technique combined with the additional benefits of efficient implementation without requiring additional sensors makes the proposed approach a suitable solution for the motion artifact removal problem for NIR studies.

## Methods

### Discrete Kalman Filtering

Kalman filtering technique uses a state space representation and least squares estimation methods for the recursive estimation of signals of interest buried within noise. Discrete Kalman filtering has been widely used in navigational and guidance systems, radar tracking, sonar ranging, satellite orbit determination, etc [9-16]. It provides an optimal estimator that processes measurements to deduce a minimum error estimate of a system by utilizing the knowledge of system ***x***_*k *_and measurement ***z***_*k *_dynamics in the form of(1)

as well as assumed statistics on system and measurement noise ***w***_*k *_and ***v***_*k*_, respectively such as being independent of each other, white and with Gaussian distributions ***w***_*k*_~ *N(0, **Q**)*, ***v***_*k*_~ *N(0,****R***). The Kalman filter is in essence a recursive solution to a least-squares problem.

If all the state space representation matrices; the transition matrix ***A ***and the output matrix ***H***; are known, the same system can be easily established and the states and the outputs can be estimated if the initial conditions are known. However, since this will be an open loop system, the estimates will not be robust. Thus, in the Kalman filter, the estimated states are obtained by using a form of feedback control where the error term obtained from the original measurements are fed back to the original system model, whose effect is determined by the Kalman gain matrix. Detailed explanation on the theory and implementation of discrete Kalman filter structure can be found in [9-16].

The final discrete Kalman filter structure [9-16] is composed of two stages of calculations: time update (predictor) equations and measurement update (corrector) equations as presented in the Appendix. The time update equations are responsible for projecting the current a posteriori state () and error covariance estimates (***P***_*k*-1_) forward in time to obtain the a priori estimates for the next time step (, ) in other words prediction of the next time step estimates. Note that a priori and a posteriori error , ***e***_*k *_and error covariance estimates , ***P***_*k *_respectively are defined as:(3)

The measurement update equations are responsible for the feedback control which incorporates a new measurement (***z***_*k*_) into the a priori estimate () through the use of optimal Kalman gain matrix (***K***_*k*_) to obtain an improved a posteriori estimate () in other words the correction of the a posteriori estimates. The optimal Kalman gain matrix ***K***_*k *_is found such as to minimize the a posteriori error covariance ***P***_*k *_in the minimum mean squares sense.

The discrete Kalman filter algorithm starts with initial estimates of a posteriori state and error covariance estimates. Once the time update equations are applied to predict a priori state and error covariance estimates of the next time step, the measurement update equations are applied to these a priori values to find their corrected a posteriori estimates at the same time step using the measurement and the optimal Kalman gain values in the feedback structure that minimizes the a posteriori error covariance matrix in the minimum mean squares sense. Then this procedure is recursively applied using the same time and measurement update pair with the newly generated a posteriori estimates in the place of initial estimates until the final time step is reached. This recursive nature makes the Kalman filter very appealing compared to other techniques (i.e. Wiener filter) since it makes practical implementations much more feasible [12].

### NIR Data Collection Protocol

In this paper, we use the data set we have collected in our previous work [4]. The protocol we had generated was composed of three types of 20 seconds of head movement periods, where the subject was asked to move his/her head up and down continuously and 20 seconds of rest periods in between the head movement periods, where subject was asked to stay still by a prompt on a computer screen. The speed of the head movements was kept constant within each of the three types of head movements, however it was gradually increased from one region to another, starting slow, then medium, then fast in order to capture the effects of different speed head movements on the NIR measurements and to test the performance of all three methods during such conditions. This procedure was repeated two times. A total of eleven subjects participated in this study. All participants signed informed consent statements approved by the Human Subjects Institutional Review Board at Drexel University.

### NIR System Used for Data Collection

**Figure 1:**
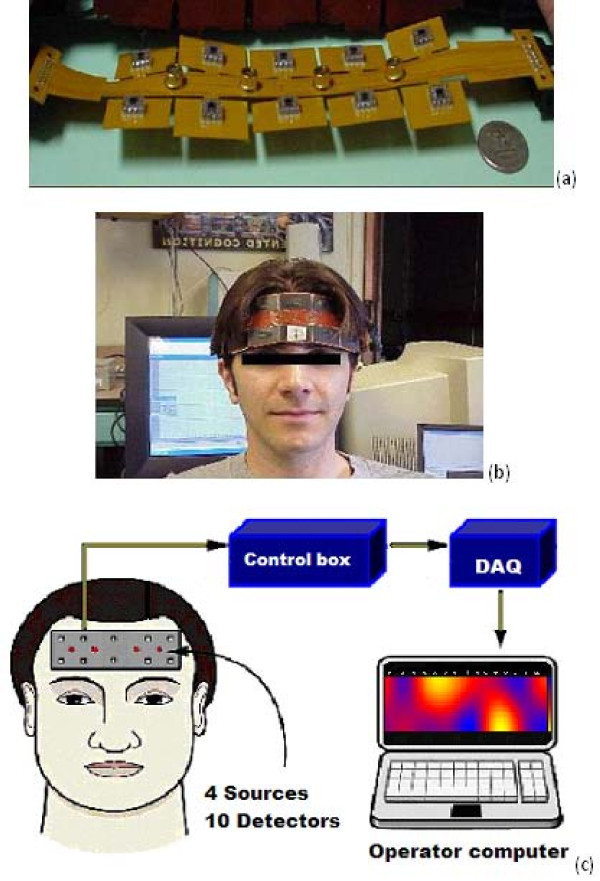
**(a) flexible NIR sensor; (b) participant wearing NIR sensor; (c) block diagram of the overall NIR system used in data collection**.

The NIR system that was used to collect the data as shown in Figure 1 was composed of an LED-based sensor that covers the entire forehead of the participant; a control module with integrated power supply for sensor control and data acquisition, and a laptop computer for the data analysis. The LED based NIR sensor was composed of four near infrared sources and ten photodiodes. The timing of firing the light sources and detectors are arranged in a way such that 16 channels of data from different places of the frontal cortex can be collected [2,3]. The raw data is sampled with a sampling frequency of 1.6 Hz.

### NIR Data Processing for the Application of Kalman, Wiener and Adaptive Filtering

In order to utilize Kalman filtering in our application, the first step was to build the system and the measurement models. We started by modeling the motion artifact free NIR signal using an autoregressive (AR) model. We estimated the AR model parameters through Yule-Walker method using one of the resting data sequence where we only have motion free brain signal. The model order was found as N = 4 by using Akaike's Information Criterion. The final AR model was then converted to a state space representation which provided the required system equations and the ***A ***matrix as given below:(5)

The measurement model was then found as:(6)

where ***z***_*k *_was the motion corrupted NIR measurement, ***x***_*k *_was the motion free NIR signal and the measurement noise ***v***_*k *_was the motion artifact. The variance of the measurement noise, the motion artifact, σ_v_^2 ^required in order to be able to perform Kalman filtering was obtained from the data regions where there is head movement. The variance of the system noise, σ_w_^2 ^is estimated using the AR model parameters and the variance of a prototype motion free NIR signal obtained during the resting period. In any real life situation, the prototypes for the noiseless NIR data and different types of motion artifact can be collected before the protocol starts. This way the variance of the motion artifact and the system model parameters can be estimated before the protocol starts.

By using the estimated system and measurement models we applied the Kalman filter to three different speed head movement data to estimate the noise free NIR signal on eleven subjects. The results were tested on NIR's one channel blood volume data for slow, medium and fast speed head movement regions in comparison with Wiener and adaptive filtering results. Note that for Wiener filtering spectral density estimates were derived from separate motion free and one trial motion corrupted data segments for each of the three motion types. The corresponding Wiener filter of each motion type was applied offline to the remaining trial region with motion artifact for noise suppression. For adaptive filtering we obtained the correlated motion data required for the technique to be applied properly using the measurements simultaneously gathered by an accelerometer attached to the forehead with the NIR sensor. This technique provided real-time application with the drawback of using an extra sensor. Detailed explanation of these practical issues in the application of these previously proposed techniques can be found in [4].

## Results and Discussion

**Figure 2:**
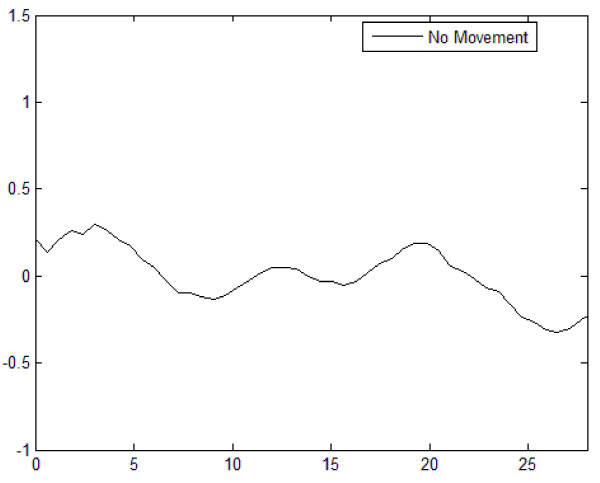
**An example motion free NIR recording**.

**Figure 3:**
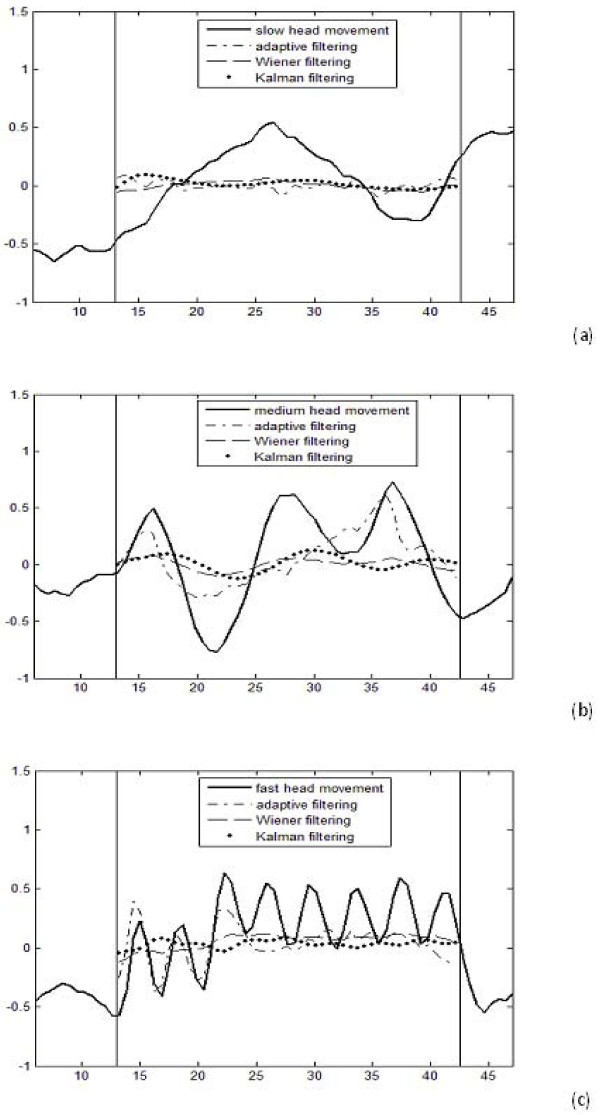
**An example Kalman filter results in comparison with adaptive and Wiener filtering outcomes for (a) slow; (b) medium; (c) fast head movement case**.

An example motion free NIR signal obtained during rest periods and outcome of the adaptive, Wiener and Kalman filtering techniques are presented in Figure 2 and Figure 3(a), (b) and 3(c) for slow, medium and fast speed head movement regions, respectively. We compared the results of these filtering approaches with the noisy NIR and rest data only in the region of interest, during the time course of the motion artifact, which is shown between the vertical lines in Figure 3. It can be easily seen from these results that the Kalman filtering algorithm successfully suppressed motion artifact in the NIR data and its results are comparable to the adaptive and Wiener filtering method. It is computationally efficient and does not require extra sensors.

In order to parametrically compare the proposed Kalman filtering technique with the previously developed adaptive and Wiener filtering techniques instead of just the visual inspection, we performed an SNR analysis to each of the algorithm results. The estimation SNR was calculated as(7)

where σ_x_^2 ^is the variance of motionless NIR data, *x(n)*, and σ_e_^2 ^is the variance of the estimation error, *e(n)*, which is the difference between the motionless NIR data and motion compensated data after filtering, (*n*), as *e(n) = x(n)-*(*n*). The input SNR was calculated as(8)

where σ_ν_^2 ^is the variance of motion artifact. Then we obtained ΔSNR = SNR_e_-SNR_i _[4] for the Kalman, adaptive and Wiener filtering results in order to show the improvements in SNRs on the estimates. A sample result of ΔSNRs for the subject whose data are given in Figure 3 are summarized in Table 1.

**Table 1:** ΔSNR (in dBs) for adaptive, Wiener and Kalman filtering for slow, medium and fast head movements

Head Movement Speed	ΔSNR (dB) (Adaptive Filter)	ΔSNR (dB) (Wiener Filter)	ΔSNR (dB) (Kalman Filter)
Slow	3.3560	5.2526	8.5055

Medium	4.1722	9.0539	7.8306

Fast	2.7906	5.7574	6.6282

In our earlier study [4], we performed a statistical analysis using all the eleven subjects data and showed that the improvement in SNR is significantly higher for Wiener filtering estimates than for adaptive filtering for all the three head movement cases. We performed the same type of analysis in order to compare the Kalman filtering results with the Wiener and adaptive filtering ones on eleven subjects. The statistical analysis results based on paired t-test comparisons are presented in Table 2. It can be deduced from these results that Kalman filtering provided significantly higher improvements in SNRs hence better estimates than the adaptive filtering in all of the three cases of head movements with no additional sensor hardware requirement. However, it did not provide significantly different SNR improvements when compared to the Wiener filter outcomes. The reason for lower SNR improvements in some cases for Kalman filtering in comparison to the Wiener approach can be due to the build up of errors as the prediction time increases in Kalman filtering, non-modeled system dynamics or the non-linearity in the system itself [13,15]. This problem can be overcome by using the backward Kalman smoother [12-15]. However, since this operation needs to be performed offline once all the data is collected, it would eliminate the real-time operation advantage of the Kalman filtering structure. The next step in our research will be to i) test these algorithms for the motion artifacts caused by the muscle movements on the forehead which can cause the direct path or ambient light to be captured by the detectors and hence result in sudden shifts in the NIR measurements and ii) analyze all of the proposed algorithms during a cognitive task.

**Table 2:** The statistical analysis results of ΔSNR (in dBs) for slow, medium and fast head movements.

Head Movement Speed	Statistical analysis for ΔSNR (Kalman vs adaptive filter)	Statistical analysis for ΔSNR (Kalman vs Wiener filter)
Slow	S. (t = 2.760, p < 0.020)	N.S. (t = -1.953, p < 0.079)

Medium	S. (t = 2.783, p < 0.019)	N.S. (t = -0.385, p < 0.708)

Fast	S. (t = 3.820, p < 0.003)	N.S. (t = -1.274, p < 0.231)

(S: Significant, N.S: Not Significant)

## Conclusions

In this paper we present a novel approach for motion artifact removal from NIR measurements using Kalman filtering. The proposed approach provides a suitable solution to the motion artifact removal problem in NIR studies by combining the advantages of the existing adaptive and Wiener filtering methods in one algorithm. The results of this preliminary study suggest that the proposed algorithm performs better than the adaptive filtering algorithm providing better SNRs while still holding the real time applicability with the further advantage of no additional sensor requirement. Our results also indicate that the proposed algorithm is comparable in SNR to Wiener filtering, without the constraints on the stationarity and with efficient real time application capability.

## Competing interests

The authors declare that they have no competing interests.

## Authors' contributions

MI conceived of the study, carried out the data processing and statistical analysis and drafted the manuscript. PC participated in the signal processing and helped in drafting the manuscript. SB participated in the study design, data collection and in drafting the manuscript. BO advised on data analysis and to draft the manuscript. All authors read and approved the final manuscript.

## Appendix

Discrete Kalman filter equations

Discrete Kalman filter time update equations

Discrete Kalman filter measurement update equations
